# Environmental Performance in the Production and Use of Recovered Fertilizers from Organic Wastes Treated by Anaerobic Digestion vs Synthetic Mineral Fertilizers

**DOI:** 10.1021/acssuschemeng.1c07028

**Published:** 2022-01-07

**Authors:** Axel Herrera, Giuliana D’Imporzano, Massimo Zilio, Ambrogio Pigoli, Bruno Rizzi, Erik Meers, Oscar Schouman, Micol Schepis, Federica Barone, Andrea Giordano, Fabrizio Adani

**Affiliations:** † Gruppo Ricicla—DiSAA, Università degli Studi di Milano, Via Celoria 2, 20133 Milano, Italy; ‡ Department of Green Chemistry and Technology, Faculty of Bioscience Engineering, University of Ghent, Coupure Links 653, 9000 Ghent, Belgium; § Alterra, Part of Wageningen UR, P.O. Box 47, 6700 AA Wageningen, The Netherlands; ∥ Acqua & Sole s.r.l., Via Giulio Natta, 27010 Vellezzo Bellini, PV, Italy

**Keywords:** ammonium sulfate, anaerobic digestion, environmental impacts, life cycle assessment (LCA), digestate, recovered fertilizers

## Abstract

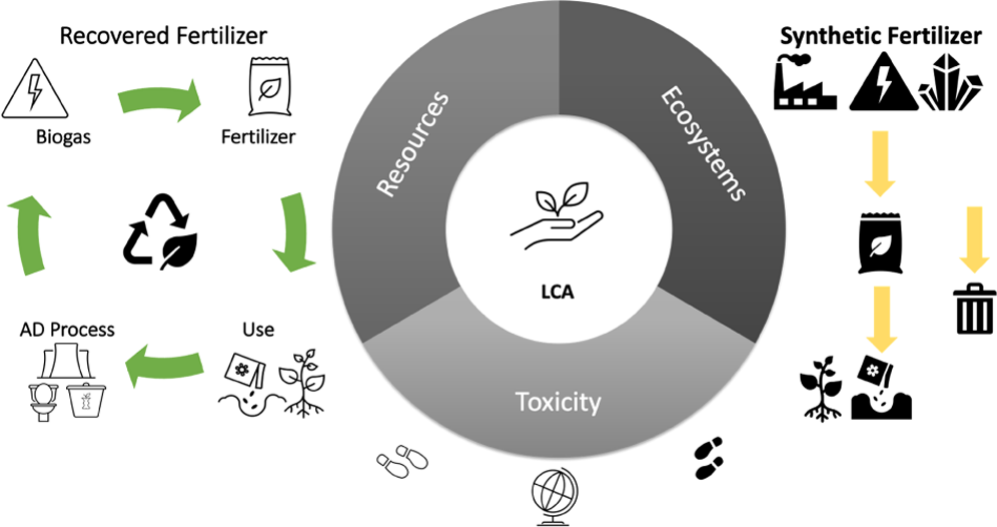

Recovered fertilizers (RFs), in the form of digestate and digestate-derived ammonium sulfate, were produced from organic wastes by thermophilic anaerobic digestion (AD) at full scale. RFs were then used for crop production (maize), substituting synthetic mineral fertilizers (SFs). Environmental impacts due to both RF and SF production and use were studied by a life cycle assessment (LCA) approach using, as much as possible, data directly measured at full scale. The functional unit chosen was referred to as the fertilization of 1 ha of maize, as this paper intends to investigate the impacts of the use of RF (Scenario RF) for crop fertilization compared to that of SF (Scenario SF). Scenario RF showed better environmental performances than the system encompassing the production and use of urea and synthetic fertilizers (Scenario SF). In particular, for the Scenario RF, 11 of the 18 categories showed a lower impact than the Scenario SF, and 3 of the categories (ionizing radiation, fossil resource scarcity, and water consumption) showed net negative impacts in Scenario RF, getting the benefits from the credit for renewable energy production by AD. The LCA approach also allowed proposing precautions able to reduce further fertilizer impacts, resulting in total negative impacts in using RF for crop production. Anaerobic digestion represents the key to propose a sustainable approach in producing renewable fertilizers, thanks to both energy production and the modification that occurs to waste during a biological process, leaving a substrate (digestate) with high amending and fertilizing properties.

## Abstract 2

Waste-recovered fertilizers can substitute synthetic mineral fertilizers promoting circular economy and reducing environmental impacts.

## Introduction

The linear economy model based on the use of fossil fuel and raw sources has led our planet to encounter major environmental problems such as climate change, land degradation, and alteration of biochemical cycles.^1^ With particular reference to N and P global flows, it has been reported that the current uses of these two elements are over Earth’s boundaries because of anthropogenic perturbation due, mainly, to fertilizer application.^2^ The use of chemically produced N and mined P is modifying and misbalancing not only the agroecosystem but also the natural ecosystems, putting biodiversity at risk.^3^

The regular production and use of mineral fertilizers in agriculture have a long track record of impacts on the environment beyond the mere addition of nutrients to the soil. Fertilizer industry production and use causes about 2.5% (1203 Tg CO_2_ equiv) of global GHG emissions,^4^ and N fertilizers account for 33% of the total annual creation of reactive N, i.e., 170 Tg N y^–1^ (fertilizers and livestock manure),^5,6^ generating big environmental problems. In addition, the production of P and K fertilizers relies upon nonrenewable and extracted resources that are becoming depleted^7^ and are concentrated (e.g., P) in only a few countries.^8^ The consequence of that is the need for new management strategies to reduce the additions of N and P into the ecosystem with particular reference to agriculture. The Circular Economy has been indicated as a new productive paradigm to produce goods, and it consists in the redesign of productive processes to allow the successive recovering of wastes for new productive processes, avoiding the use of new resources.^9^

Organic wastes can be explored as raw materials to recover nutrients and organic matter, representing an example of Circular Economy. To do so, wastes should be accurately chosen so that nutrient recovery can be made by applying suitable technologies,^10^ producing fertilizers to replace synthetic ones.^11^ Anaerobic digestion (AD) is a suitable biotechnology for producing biofertilizers, thanks to the process that modifies organic matter and the nutrients it contains, resulting in a good amendment and fertilizer properties of the end product, i.e., digestate.^12−14^ In addition, the AD process renders the digestate more suitable for subsequent biological/physical/chemical treatments allowing organic matter (OM) and N and P to be separated, producing both an organic amendment and N and P fertilizers.^10,15−17^

The recovery of nutrients allows the production of fertilizers able to substitute for synthetic ones, thus reducing the necessity to produce fertilizers using fossil energy (N and P) and fossil resources (P and K),^18^ and closing nutrient cycles. In addition, the recovery, also, of the organic matter represents a solution to the problem of low organic matter (OM) content (<1%) of soils,^19^ which are attributed to the high carbon dioxide emissions which result from the intensification of agricultural practices.^20^

Despite the clear need to better manage nutrients already present in the ecosystem without adding new ones, a significant obstacle to this is the low efficiency and environmental performance, which have been attributed to recovered nutrients.^5,21^ Synthetic fertilizers contain concentrated nutrients under available forms, and so they are easy to apply to meet crop requirements. By contrast, the recovered wastes (sewage, manure, digestates, etc.) contain nutrients with low efficiency and low concentration, and which also require good practices to be used to avoid environmental impacts.^22,23^ Low-nutrient use efficiency (NUE) of recovered fertilizers might be due to their nonappropriate chemical form (mineral vs organic forms), loss as NH_3_ volatilization (10–65%), NO_3_^–^ leaching and runoff (1–20%), and nitrification–denitrification (1–30%).^24,25^ Therefore, the increase of NUE and environmental outcomes of recovered fertilizers represent challenges for modern agriculture.^26^

Recently, a scientific paper described,^10^ at full scale, an AD plant producing recovered fertilizers (renewable fertilizers—RF) by anaerobic digestion, proposing that these fertilizers be used to substitute completely for fertilization by synthetic mineral fertilizers (SFs). Despite this, producing recovered fertilizers does not mean that they are capable of replacing synthetic ones ensuring better environmental performance.^27^ Therefore, the assessment of environmental impacts of recovered fertilizers needs to be studied in comparison with synthetic ones using an appropriate approach, i.e., life cycle assessment (LCA) fed by validated data (full-field data). To do this, the entire supply chain, i.e., production of fertilizers and their use in place of synthetic ones, must be considered.

The literature over the past 10 years has focused on renewable energy production through anaerobic digestion and less on the analysis of impacts related to the recycling of nutrients and their use. For example, Hijazi et al.^28^ reviewed 15 LCAs related to anaerobic digestion of different biomasses focusing on the amount of renewable energy produced, used as a functional unit, rather than on recycling of nutrients, similar to other studies.^29,30^

On the contrary, Timonen et al.^31^ underlined the importance of AD in recovering nutrients, and LCA performed considered digestate production (renewable fertilizer) together with energy. The authors compared three different digestate management to consider a multifunctional approach (bioenergy and fertilizers) capable of increasing the efficiency of the agricultural system. The work done underlined that the emissions due to the storage of digestate, its transport, and use in the open field were higher than those due to the use of chemical fertilizers. Despite this, by combining the emissions due to anaerobic digestion and those due to the use of digestate, emissions were lower than those due to the production and use of mineral fertilizer. In the work cited, similarly to others,^27,30,32^ data used (e.g., ammonia and N_2_O emissions and nitrate leaching) were calculated using IPCC coefficients and no experimental data were used. Lyng et al.^33^ underlined the importance of direct measurement from full-scale realities to avoid over- or underestimates.

This work likes to contribute to the existing literature by providing novelty in terms of the LCA approach able to consider the whole chain in producing and using recovered fertilizers from sewage sludge by AD. In particular, this work aims to complete the path of the proposed Circular Economy in agriculture, by measuring directly in the open field, the impacts derived from the use of recovered fertilizers, used according to virtuous approaches capable of reducing the resulting impacts. The full-scale approach and the use of directly measured data aim to correctly and experimentally evaluate the effectiveness and sustainability in the recycling of nutrients to replace synthetic mineral fertilizers.

## Materials and Methods

### Goal and Scope

LCA analysis aims to measure the environmental impacts related to both production and to subsequent agronomic use of digestate and ammonium sulfate (recovered fertilizer) (RF) produced by the anaerobic digestion process using a mix of organic wastes (Scenario RF), compared to the production and use of synthetic fertilizers (SFs), i.e., urea, triple phosphate, and potassium sulfate (Scenario SF). This study covered the entire production and use of fertilizers, i.e., “from cradle to grave”^34^ as it analyzed a large full-scale anaerobic digestion plant used to transform organic wastes into biofertilizers (production phase),^10^ and the subsequent full-field application of the recovered biofertilizers (digestate and ammonia sulfate).

### Functional Unit

The functional unit (FU) provided a reference to which all data in the assessment were normalized. Because this study considered the impacts derived from the production and use of fertilizers on maize crop, the functional unit chosen was referred to the fertilization (fertilizers production and use) of 1 ha of maize, i.e., for the Scenario SF: 402 kg of urea (185 kg of N), 476 kg of chemical ammonium sulfate (100 kg N), 195 kg of triple phosphate (89 kg of P_2_O_5_), and 165 kg of potassium sulfate (82.5 kg of K_2_O), and for Scenario RF: 48 Mg of digestate, i.e., 370 kg of total N, i.e.,185 kg of effective N, 317 kg of P_2_O_5_, and 43 kg of K_2_O, 1.38 Mg of recovered ammonium sulfate (100 kg of N), and 80 kg of potassium sulfate (40 kg of K_2_O) (see Table 1).

**Table 1:** Inventory Data of the Considered Scenario

	unit	quantity	data source
input			
waste input (total)	Mg y^–1^	81 886	provided by facility^a^
methane (from national grid)	sm^3^ y^–1^	228 177	provided by facility
water (from aqueduct)	m^3^ y^–1^	19 744	provided by facility
water (from well)	m^3^ y^–1^	14 044	provided by facility
water (total)	m^3^ y^–1^	33 788	provided by facility
electricity consumed from the grid	kWh y^–1^	7189	provided by facility
sulfur acid	Mg y^–1^	316	provided by facility
output			
digestate produced	Mg y^–1^	112 322	provided by facility
electricity produced and fed to the grid	kWh y^–1^	5 349 468	provided by facility
electricity produced and reused in the process	kWh y^–1^	2 395 215	provided by facility
total electricity produced	kWh y^–1^	7 737 494	provided by facility
ammonium sulfate	Mg y^–1^	571	provided by facility
wastes from sieving sent to landfill	Mg y^–1^	2.5	provided by facility
biogas produced	Mg y^–1^	3842	provided by facility
thermal energy produced (by CHP)	MWh_th_ y^–1^	5976	provided by facility
emissions (from distribution) digestate			
ammonia (N-NH_4_)	kg ha^–1^	25.2	detected on-site by the authors^b^ (Table S4)
direct dinitrogen monoxide (N-N_2_O)	kg ha^–1^	9^c^	detected on-site by the authors (Table S4)
indirect dinitrogen monoxide (N-N_2_O)	kg ha^–1^	0.8	IPCC 2006
nitrate leaching (N-NO_3_)	kg ha^–1^	83^d^	IPCC 2006
NO*_x_* (N-NO_*x*_)	kg ha^–1^	0.5	IPCC 2006
P surface run off (P)	kg ha^–1^	1.4	EDIP 2003
urea			
ammonia (N-NH_4_)	kg ha^–1^	25.2	detected on-site by the authors (Table S4)
direct dinitrogen monoxide (N-N_2_O)	kg ha^–1^	9^b^	detected on-site by the authors (Table S4)
indirect dinitrogen monoxide (N-N_2_O)	kg ha^–1^	0.8	IPCC 2006
nitrate leaching (N-NO_3_)	kg ha^–1^	83^c^	IPCC 2006
NO*_x_* (N-NO*_x_*)	kg ha^–1^	0.3	IPCC 2006
carbon dioxide (C-CO_2_)	kg ha^–1^	80.2	IPCC 2006
P surface run off (P)	kg ha^–1^	0.2	Nemecek and Kägi 2007
use of nutrients			
RF^e^			
digestate	Mg ha^–1^	48	data from authors^f^
TN supplied by digestate	kg ha^–1^	370	data from authors
TN delivered by ammonium sulfate	kg ha^–1^	100	data from authors
P supplied by digestate	kg ha^–1^	138	data from authors
K supplied by digestate	kg ha^–1^	36	data from authors
K delivered as potassium sulfate	kg ha^–1^	34	data from authors
SF^c^	kg ha^–1^		
TN supplied by urea	kg ha^–1^	185	data from authors
TN delivered by ammonium sulfate	kg ha^–1^	100	data from authors
P provided by triple phosphate	kg ha^–1^	39	data from authors
K supplied as potassium sulfate	kg ha^–1^	70	data from authors

a Provided by facility: data acquired directly from the full-scale plant under study.

b Detected on-site by the authors: data acquired from open-field experimentation (see also the SI and Table S4).

c N_2_O emissions were considered similar (calculated on 1 ha surface) for the two Scenarios as revealed by full-field measurements made after digestate and urea distribution (see Table S4).

d N leaching was assumed similar (calculated on 1 ha surface) for the two Scenarios as revealed by soil sampling made at 1 m soil depth in full-field trials (see Table S4).

e RF: recovered fertilizer Scenario and SF: synthetic fertilizer Scenario.

f Data from authors: data derived from fertilization plan and fertilizer properties (see Tables S1–S3).

### System Description

#### Anaerobic Digestion Plant

**Figure 1:**
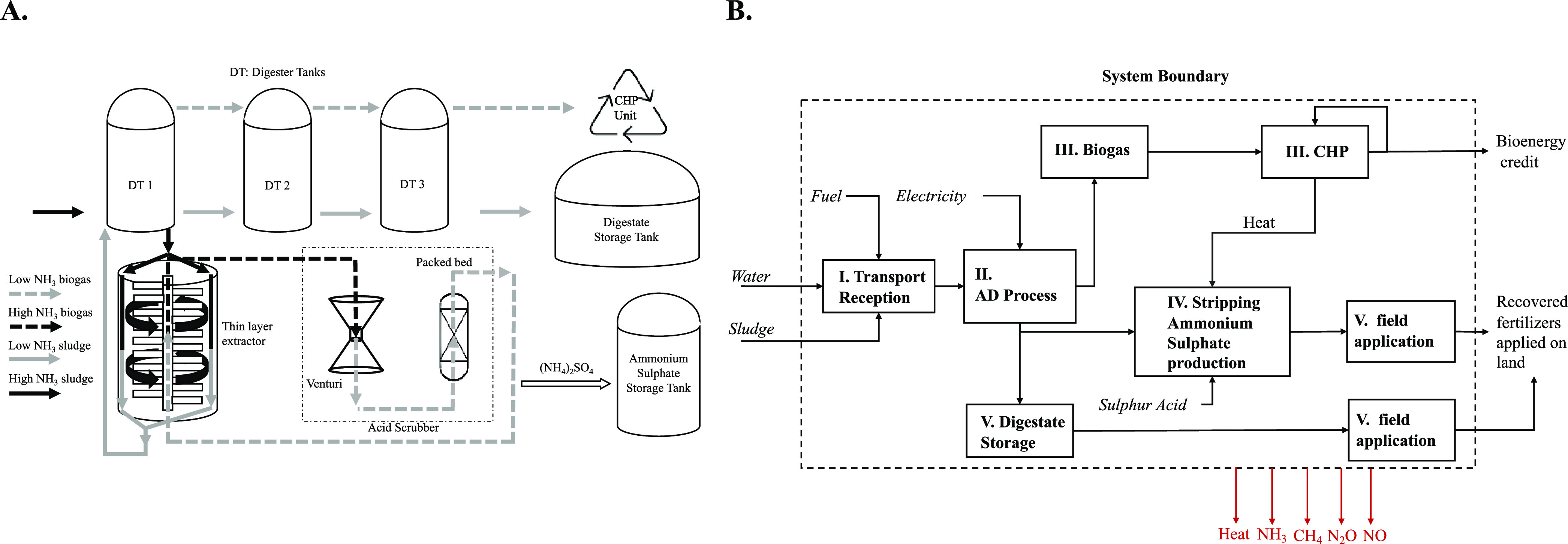
Anaerobic digestion (AD) plant and nitrogen-stripping unit layouts (a); system boundaries and main processes for the recovered fertilizers (RFs) (b).

The AD plant (1 MWe power) for the combined production of fertilizers and energy is situated in the Lombardy Region (North Italy).^10^ The plant exploits anaerobic digestion (AD) to transform different organic wastes (sewage sludges produced by municipal WWTP, agri-food factories, and liquid pulp-fraction of source-separated domestic food wastes) into organic-mineral fertilizers, i.e., digestate, mineral N-fertilizer (i.e., ammonium sulfate), and energy (thermal and electrical). The plant is composed by two main sections comprising the AD plant and the ammonia-stripping unit (Figure 1a).

The AD plant produces biogas that is exploited to produce electrical energy delivered to the national grid and is also used for plant autoconsumption and heat that is used for digester heating by steam injection and in the ammonia-stripping unit. During the process, several data were continuously monitored: digestate, pH (daily), digestate temperature, produced biogas, and biogas composition (CH_4_, CO_2_, and H_2_S, this latter four measurement per day).

Anaerobic digestion takes place in three reactors, working in series, of 4500 m^3^ each, made in carbon steel, with an average hydraulic retention time (HRT) of 45–50 days, which is longer than usual HRT for AD plant-treating sewage sludge but useful to ensure good biological stability and sanitation.^10^ The AD process is performed in thermophilic conditions (55 °C), where the temperature is kept stable using the heat produced from the combined heat and power (CHP) unit. Reactor tanks have no mechanical mobile parts inside, with digestate mixing guaranteed by a system of external pumps. The tanks are covered with a gasometric dome membrane and maintained at constant pressure. The system withdraws digestate from the second digester tank (DT 2) (Figure 1a) to the thin layer extractor, where ammonia is stripped from digestate using the biogas or air.^10,35^ The thin layer extractor consists of a cylindrical tank having inside a rotor with radial paddles, which by rotating at high speed keeps the digestate spread in a thin layer (few millimeters thick) on the internal walls of the cylinder.

Meanwhile, the rotor keeps biogas at high turbulence to enhance the exchange of ammonia from the digestate to the gas. The transfer of ammonia occurs in a counter current; the digestate is pumped into the top of the cylinder, and it goes down by gravity in a thin layer while gas flux is from the bottom to the top. The walls of the cylinder are warmed at 80 °C to increase the exchange from the digestate to the gas which is injected at 70 °C. After the stripping in the thin layer, the low-content ammonia digestate is pumped back to the first digester (DT 1) while carrier gas in a closed-loop cycle goes to the acid scrubber unit, where ammonia reacts with sulfuric acid-generating ammonium sulfate. Both recovered fertilizers produced were used in substitution for synthetic fertilizers, both at presowing (digestate) and as top-dressing (ammonium sulfate).

#### Recovered Fertilizers Produced

Recovered fertilizers (renewable fertilizers) characteristics are listed in Tables S1 and S2; a complete description can be found in Pigoli et al.^10^ The previous characterization made also included organic contaminants and target-emerging organic contaminants (Table S1).

#### Full-Field Agronomic Use of Renewable Fertilizers in Substitution of Synthetic Mineral Fertilizers

Full-field agronomic performance and impact measurements, i.e., air emissions (NH_3_, N_2_O, CH_4_, and CO_2_) and nitrate leaching, were carried out on soil plots distributed randomly close to the AD plant. Digestate was injected into the soil at a depth of 15 cm at the dose required assuming a N efficiency of 0.5, as suggested by the Regional Plan for Water Protection from Nitrate from Agriculture.^36^ For the SF Scenario, urea was spread onto the soil surface following a routine agricultural procedure. The dosage of fertilizers was made according to common practices. Fertilizers used, doses applied, and spreading methodology are reported in detail in Table S3 in the Supporting Information and summarized in Table 1.

#### Emissions

GHG emissions (N_2_O, CH_4_, and CO_2_) were measured in 2020, following the entire agronomic season of maize: from May (sowing) to October (harvest). The determination of emissions was conducted through the use of non-steady-state chambers.^37^ Sampling chambers were placed in each of the experimental plots; furthermore, to obtain a background measurement, another three chambers were placed on nonfertilized plots. The air sampling inside the chamber was carried out with a frequency of 1–8 times a month, depending on the season and the state of the crop. The air taken was then analyzed in the laboratory using a gas chromatograph, according to the method reported by Piccini and colleagues.^38^ The cumulative emissions were obtained by estimating the flows in the nonsampling days, by linear interpolation.^39^ The concentration of NH_3_ was monitored by the exposure of α passive samplers.^22,40^ For each plot, α samplers were installed in sets of three. To obtain background environmental concentration values, an additional sampling point was placed at a distance of about 1000 m away from fertilized fields and other possible point sources of NH_3_ emissions.

### System Boundaries and Data Inventory

#### System Boundaries

The system boundary starts from the organic waste collection and transport encompasses the production of digestate/biofertilizer and ammonia sulfate, the correlated processes for producing biogas which is transformed into electric energy and thermal energy and finally the use of the digestate in the field. The system boundary is represented by the dashed line in Figure 1b and comprises five main processes for Scenario RF (recovered fertilizer): (i) the transport of sludge and organic wastes to the AD plant (assuming 100 km on average), (ii) the AD process, (iii) the biogas combustion and electricity production in CHP, (iv) the digestate stripping process and ammonium sulfate production, and (v) the digestate storage, handling, and distribution into fields. Capital goods were included in the system, considering a lifespan of the structure of 20 years. The Scenario SF (synthetic fertilizer) encompassed the production of urea, triple phosphate, and potassium sulfate fertilizers (including logistics and transportation) and the timely distribution on fields. This Scenario was modeled using data coming from the literature and databases (Ecoinvent 3.6).^41^

The main data inventory is reported in Table 1; inputs and output of production were all taken directly from the plant facility. Air emission of the two systems, i.e., ammonia, methane, nitrous oxide, and carbon dioxide, was measured directly on monitored field plots as previously reported (Tables 1 and S4). Indirect dinitrogen monoxide and NO_*x*_ were estimated according to IPCC.^42^ Nitrate leaching was calculated according to IPCC^42^ for the Scenario SF, based on the N distributed, and assumed to be equal for Scenario RF, as the monitoring of nitrate content in deep soil layers during the year showed no differences (Table S4). Phosphorus in soil, leaching, and run off was modeled according to Ecoinvent report 15.^43^ Heavy metals supplied were included in the model according to the characterization data of digestate, plant uptake, and accumulation rate in the soil system.^44,45^ The input of organic pollutants was considered for PCDD/F, DEHP, and PAH contained in digestate, as a proper numerical quantification was workable (see Table S1).

### Modeling Framework and Approach to Multifunctionality

The modeling framework of this study was attributional, i.e., digestate and ammonium sulfate were considered as the target products of the production chain. Biogas was produced and valorized in the CHP module to generate electricity and heat. To consider these outputs and to make the two systems (Scenario RF and Scenario SF) comparable, the approach of system substitution, i.e., crediting for the avoided burden, was chosen. The option of system substitution was not exploited to include the service of waste treatment (i.e., incineration or landfill) that is performed, as it would have introduced great variability in the credits of the service. This approach was very prudential, as it did not consider the alternatives for disposal of organic wastes that in any case would be necessary and impacting. However, the credits for renewable electricity were accounted for and considered for substituting the electricity mix distributed in the national grid.

### Life Cycle Impact Assessment

The life cycle impact assessment (LCIA) was based on the emissions and resource inputs identified during the data inventory, which was processed into indicators that reflect resource shortage and environmental burdens. The software SimaPro Analyst 9.1.1.7^46^ was used for the computational implementation of the inventories and the set of libraries covered by Ecoinvent databases v3.6, 2019 to analyze environmental impacts. Because of its representativeness at the global scale, the ReCiPe 2016 method (version 1.13),^47^ which contains midpoint impact indicators and end point areas of protection, was used to assess the environmental performance of biofertilizers and energy production. Global normalization factors from the same method were used.^48^

The robustness of the LCA results was assessed by Monte Carlo analysis, setting 10 000 runs.^49^

## Results and Discussion

**Figure 2:**
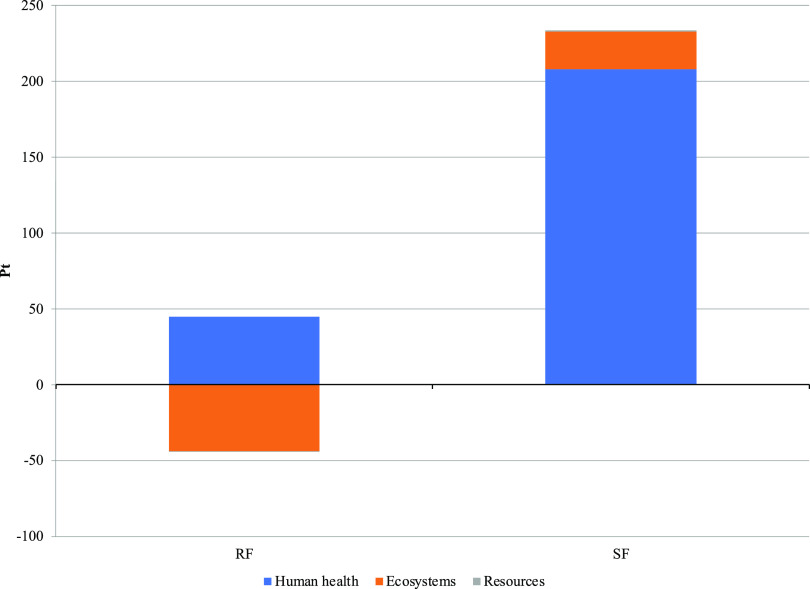
Comparative environmental results for Scenarios Recovered Fertilizers (RFs) and Synthetic Fertilizers (SFs). Impact assessment (Ecopoint—Pt) calculated according to the ReCiPe 2016 end point (H) V 1.03 impact assessment method.

The results of the two Scenarios reported as midpoint indicators and split for fertilizers production and use, as well as the impact deviations taking as reference Scenario RF, are shown in Table 2. The Scenario RF showed better environmental performances than the system encompassing the production and use of urea and commercial fertilizers (Scenario SF). In particular, for the Scenario RF, 11 of the 18 categories showed a lower impact than in the Scenario SF, and four of the categories (ionizing radiation, terrestrial ecotoxicity, fossil resource scarcity, and water consumption) showed net negative impacts in the Scenario RF, getting the benefits from the credit of renewable energy production by AD. The final end point single score ranked 48 and 215 points for the Scenario RF and Scenario SF, respectively, which summarizes the globally better outcome of the Scenario RF (Figure 2). Analysis and contributions of the processes to the categories are discussed below.

**Table 2:** Impact Category Values for the Two Compared Systems SF and RF with Their Respective Contribution Due Production and Use (Field Emission and Distribution), and Credit Related for the Electricity Generated (CRE)^a^

		RF				SF		
impact category	unit	production	use	CRE	total	production	use	total
global warming	kg CO_2_ equiv	669	3999	–1315	3354	834	3966	4800
stratospheric ozone depletion	kg CFC11 equiv	0	0.1	0	0.1	0	0.1	0.1
ionizing radiation	kBq Co-60 equiv	38	10	–204	–156	82	4.5	86
ozone formation, human health	kg NO_*x*_ equiv	5	2	–3	4	1	1.0	2
fine particulate matter formation	kg PM2.5 equiv	2	6	–2	7	1	6.2	8
ozone formation, terrestrial ecosystems	kg NO*_x_* equiv	5	2	–3	4	1	1.0	2
terrestrial acidification	kg SO_2_ equiv	6	50	–5	51	4	50	54
freshwater eutrophication	kg P equiv	0.1	8.4	–0.3	8.2	0.3	0.2	0.5
marine eutrophication	kg N equiv	0	17	0	17	0.0	17	17
terrestrial ecotoxicity	kg 1,4-DCB	1247	240	–1370	117	2550	114.8	2664
freshwater ecotoxicity	kg 1,4-DCB	8	351	–11	348	13	0.6	14
marine ecotoxicity	kg 1,4-DCB	12	492	–16	488	23	0.9	24
human carcinogenic toxicity	kg 1,4-DCB	35	9	–25	19	19	1.4	20
human noncarcinogenic toxicity	kg 1,4-DCB	266	54 585	–330	54 521	458	88.8	547
land use	m^2^ a crop equiv	7	3	–4	6	6	1.1	7
mineral resource scarcity	kg Cu equiv	3	1	–1	4	9	0.4	9
fossil resource scarcity	kg oil equiv	134	27	–384	–224	313	16	329
water consumption	m^3^	631	189	–8575	–7755	1196	86	1282

a Impact assessment calculated according to ReCiPe 2016 Midpoint (H) V.1.1. FU: 1 ha Maize.

### Midpoint Results of Impact Categories Related to Ecosystem Quality

#### Global Warming Impact Category

The production of the recovered fertilizers (Scenario RF), which included sludge transport and handling, the AD process, ammonia stripping, and biogas burning, without considering the electricity credits, caused the emission of 669 kgCO_2equiv_, lower than the data reported for the production of synthetic mineral fertilizers, i.e., 834 kgCO_2equiv_. Beyond, thanks to the credits (avoided CO_2_ emissions) due to the production of renewable energy (biogas), the value of the fertilizers production was negative, i.e., −646 kgCO_2equiv_. With reference to the fertilizer use, which was reported to be the critical point in terms of emissions and environmental impacts for the recovered fertilizers,^50^ the impact for the Scenario RF (i.e., 3999 kgCO_2equiv_) was only slightly higher than that for the Scenario SF (i.e., 3966 kgCO_2equiv_) because of the higher energy consumption needed for digestate distribution into the soil than that required for urea and other mineral fertilizers distribution (Scenario SF).

From the data reported above, it was derived that the total net impact measured for the production and use of RF was of 3354 kgCO_2equiv_, with this figure being lower (−30%) than that calculated for the Scenario SF, i.e., 4800 kgCO_2equiv_ (Table 2). GHG impacts were due above all to direct emission of N_2_O coming from nitrogen dosed to the soil as fertilizers, with the GHG coming from biogas burning and mass transportation playing only a minor role. The impacts measured for this gas were the same for the two Scenarios studied, since the measured N_2_O emissions were not significantly different from each other (Table S4).

Results of this work appear more interesting if it is considered that to add an equal quantity of efficient N to the two Scenarios, much more N was added to the soil in the Scenario RF, i.e., total N of 370 kg ha^–1^ (370 kg ha^–1^ × 0.5 = 185 kg ha^–1^) (Table S3) than in the Scenario SF, i.e., 185 kg ha^–1^ of N, suggesting that only the efficient (mineral) fraction of total N was responsible for N_2_O emission, since these two figures were identical for the two Scenarios studied (i.e., total mineral N dosed of 185 and 185 kg ha^–1^ of N for Scenarios RF and SF, respectively) and that organic N (contained in the digestate) appeared not to additionally contribute at to emissions.

This result was consistent with the high biological stability of the digestate, measured by potential biogas production (BMP) (Table S1), that was even lower (i.e., with higher biological stability) than those reported for well-matured composts,^51^ leading to null or a very low rate of mineralization of the organic N in short-medium time. The biological stability of the organic matter has recently been reported to play an important role in defining N mineralization in soil. Tambone and Adani^52^ reported that mineral N produced during organic substrate incubation correlated negatively with CO_2_ evolved during soil incubation, i.e., the more stable was the substrate, the less C (and N) mineralization occurred. In this work, CO_2_ and CH_4_ measurements carried out directly on plots during the cropping season (Table S4) indicated the absence of differences in C emission for soil fertilized with synthetic fertilizers and digestate but also with the control (no fertilizers added) confirming that organic matter added with digestate was stable, contributing to restore soil organic matter. The increase of total organic carbon (TOC) in soil treated with digestate after 3 years of fertilization, compared to soil fertilized with mineral fertilizers, seems to confirm this fact (TOC increased after 3 years from 10.3 ± 0.6 g kg^–1^ dry weight (dw) to 12.3 ± 0.4 g kg^–1^ dw, differently from the mineral fertilized and unfertilized plots that did not show any increase) (unpublished data).

Results obtained in this work differed from those of previous studies that reported higher emissions of N_2_O when recovered fertilizers (digestate) replaced mineral fertilizers.^32^ Nonetheless, in that case, N_2_O emissions were assumed (not measured directly) to be of 1% of the total N from mineralization, mineral fertilizers, digestate, and existing crop residues; in addition, no data regarding the OM quality of digestate (potential N mineralization), i.e., biological stability, were reported. It can be concluded that N_2_O emissions depended on available N (mineral) plus the easily mineralizable fraction of the organic N, which depended, in the first instance, on the biological stability of the organic substrate, so that this parameter becomes important for a rough estimation of the potential N_2_O emission. This result was in contrast with that reported in the literature which indicated a direct proportionality between the total amount of nitrogen supplied and N_2_O emissions,^42,53^ without any specification of N type, i.e., organic vs mineral N and organic matter stability responsible for potential N mineralization. We consider that this approach could lead to a misinterpretation of the real impacts of recovered organic fertilizers that need, as already discussed, to be better characterized.

Ammonia emissions represent another important issue in determining environmental impacts when using fertilizers. The full-field approach indicated that there were no differences in ammonia emissions between Scenario RF and Scenario SF (Table S4) thanks to the digestate injection that resulted in a strong mitigation in ammonia emissions in comparison with superficial spreading,^23^ as also confirmed by the literature.^22^ The low ammonia emissions did not increase N_2_O emission, as already discussed, in contrast with what has been reported in the literature, i.e., that ammonia emissions abatement led to an increase in N_2_O emissions,^54^ indicating that a well-stabilized organic substrate and the adoption of an efficient distribution technique allowed containment of both NH_3_ and N_2_O emissions. The high biological stability of the digestate, providing for low organic matter mineralization, limited, also, the NO_3_^–^ leaching for the Scenario RF, which was, according to the data measured directly at the full field during the crop season, not significantly different from that measured for the Scenario SF (Table S4).

#### Other Impacts

The identical N_2_O emissions reported for the two Scenarios studied led, also, to similar stratospheric ozone depletion impact, since the emissions of ozone-depleting substances (ODSs) are mainly due to direct N_2_O emissions from fields.

Ionizing radiation quantified the emission of radionuclides in the environment that may be due to nuclear activity, but also to fuel burning. The Scenario RF achieved a total negative impact because of the production of renewable electricity that compensated for the other emissions caused by transport (transport of sludge to the AD facility), digestate handling, and distribution. Considering just the fertilizer use, the measured impact was higher for the Scenario RF than that for the Scenario SF, i.e., 9.7 vs 4.5 kBq Co-60_equiv_, (Table 2). High water content and low-nutrient concentration for digestate, leading to more energy consumption for its distribution than for synthetic mineral fertilizers, were responsible for the higher impact.

**Figure 3:**
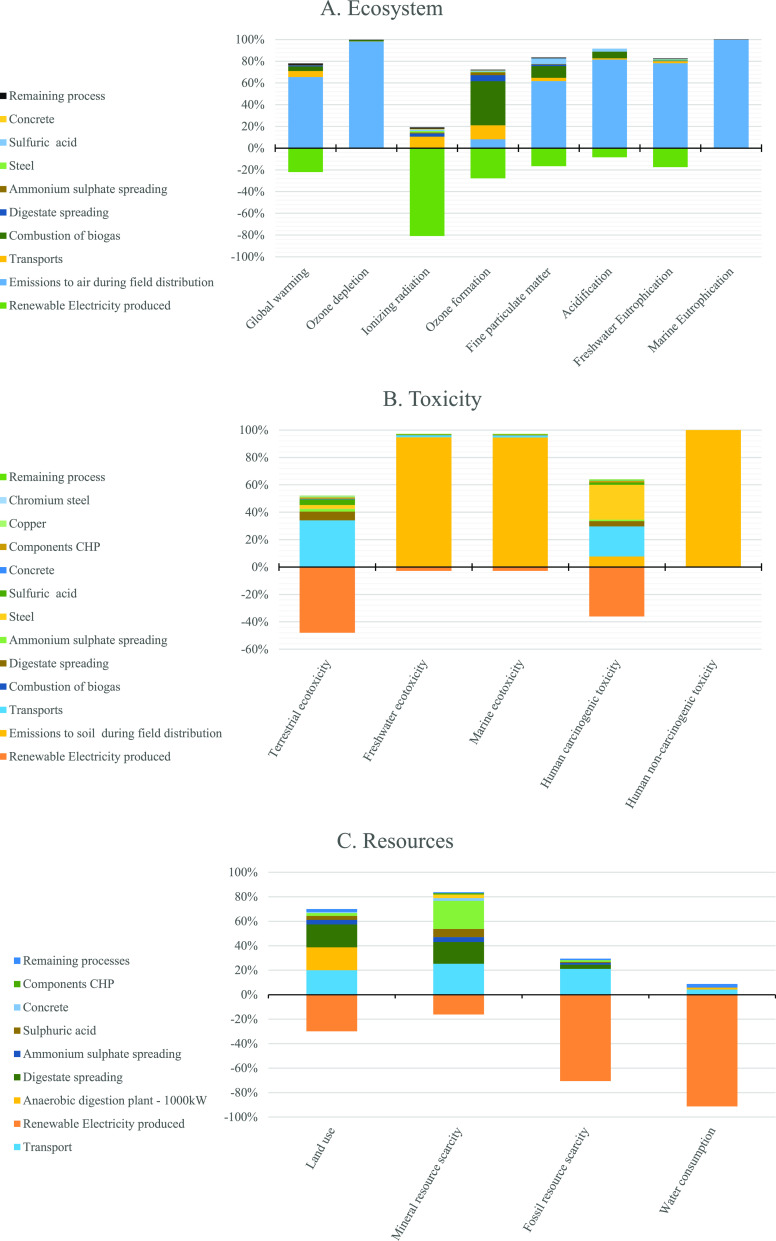
Process contribution to the impact categories of the Scenario RF, focusing on the ecosystem (a), toxicity (b), and resources (c). Impact assessments were calculated according to the ReCiPe 2016 midpoint (H) V 1.03 method and data reported as percent of the total impact.

The categories ozone formation (human health and terrestrial ecosystem) that quantified the potential molecules leading to the formation of ozone as NO*_x_* equivalent^47^ were two of the six categories reported to be higher for the Scenario RF than the Scenario SF, the main contributor to this category being the biogas combustion for electricity production (Figure 3a). Less important, i.e., about 10%, was the impact due to direct emissions in the field, i.e., distribution of digestate (fuel machinery) and distribution of ammonium sulfate and NO*_x_* direct emissions from land.

Impact due to fine particulate matter formation was almost identical for the two Scenarios (Table 2). This result was because this impact was generated mostly by ammonia emissions during field fertilization, which was similar for the two Scenarios investigated (Table S4). Particulate matter due to biogas burning in the CHP unit (producing both heat and electricity), fuel combustion for sludge transport to the plant, and digestate field distribution were balanced by credits due to renewable energy produced, determining only a slightly lower value than that calculated for the Scenario SF.

Terrestrial acidification, which is related to nutrients supplied, i.e., deposition of ammonia, nitrogen oxides, and sulfur dioxide in acidifying forms, displayed similar values for the Scenario RF and Scenario SF (Table 2). Scenario RF had a slightly higher impact due to fertilizer distribution because of NO_*x*_ emissions related to the greater use of machinery necessary for the distribution of digestate. Previous studies reported opposing results, i.e., an increase in potential acidification when N mineral fertilizer was replaced by digestate.^32,55^ On the other hand, when the use of proper timing and distribution techniques were considered, previous LCA results were in line with those of this work.^56,57^

Freshwater and marine eutrophication deal with the increase of nutrients (namely P and N), leading to excessive primary productivity and finally biodiversity losses. Freshwater eutrophication (expressed as P equivalent) displayed a higher value for the Scenario RF than the Scenario SF because the total amount of P brought to the soil by digestate was greater than the crop requirement and so higher than P dosed in the Scenario SF. Phosphorus overdose depended on the N/P ratio that determined an excess of P when dosing the correct amount of efficient N required by a crop (Table S3). N/P ratio imbalance is well known and documented for animal slurries and digestates,^58^ and it is even more accentuated in the case of digestates produced by sewage sludge, in which the previous wastewater purification process mainly determines an accumulation of P, while the denitrification processes displace part of the nitrogen.^59^

For marine eutrophication, the impact measured for the two Scenarios was equivalent, as the N leached assessed in full-field trials was recorded as equal for the two Scenarios studied (see Table S4, Supporting Information).

### Midpoint Results of Impact Categories Related to Human Health Protection

The inclusion of toxicity categories (USEtox) (Table 2) in the ReCiPe 2016 methodology allowed us to better focus the impacts of the production and use of fertilizers when compared with previous work done that considered only the main agricultural-related indicators, such as global warming potential, eutrophication, and acidification.^32,57^

The use of fertilizers determined a higher impact for the Scenario RF than the Scenario SF for the toxicity categories, i.e., Freshwater and marine ecotoxicity and human noncarcinogenic toxicity, because of heavy metals (HM) (above all Zn) supplied to soil with digestate. This figure has already been highlighted in literature for other organic fertilizers (pig slurries) because of their very high Zn and Cu contents.^60,61^ In particular, the amount of Zn applied to the soil with the digestate corresponded to 3.8% of that present in the 15 cm of surface soil, but after 3 years of experimentation, no differences were observed in soil Zn content (Table S5). Nevertheless, analyzing grains, higher Zn content was revealed for plot amended with digestate (Table S5) although the same grain production was measured (Table S6). However, this content was in line with those reported in the literature for both maize grain and other cereals (i.e., rice and wheat).^62^

Further effort should be made to decrease impacts, reducing HM in sewage sludge by selecting the cleanest ones. The terrestrial ecotoxicity impact was mainly generated during the fertilizer production (Table 2); in particular, for the Scenario RF, the impact was due above all to the transport of sludge to the AD plant (Figure 3b), while for the Scenario SF, it was the N fixation process (ammonia steam reforming) that determined the impact. Nevertheless, the Scenario RF benefitted from the production of electricity, significantly reducing the impacts. Finally, the category human carcinogenic toxicity also showed a better environmental outcome for the Scenario RF than the Scenario SF, thanks to the credits from the production of renewable energy (Figure 3b).

### Midpoint Results of Impact Categories Related to Resource Scarcity Protection

The use of both renewable energy (biogas) and recovered material (sewage sludge) to produce fertilizers (digestate and ammonia sulfate) led, also, to high efficiency in terms of land use, mineral resource use, fossil resources, reducing, until negative, these impacts (Table 2).

### Single End Point Indicator

The single end point indicator provided by the ReCiPe method allows one to view the normalized and weighted impacts in a synthetic manner and is divided into the three areas of protection, i.e., ecosystem, toxicity, and resources (Figure 2). The Scenario RF was significantly better than the Scenario SF, and in particular, the indicators showed for the Scenario RF, not only an impact reduction but also the prevention of impact in the areas of protection of resources and human health, as previously reported.^27,63−66^

### Further Scenarios Reducing Environmental Impacts in Producing and Using Renewable Fertilizers

Life cycle assessment is a powerful tool for describing impacts due to fertilizer production and use, highlighting positive and negative effects for renewable fertilizers vs synthetic mineral fertilizers in a real case study. However, LCA is also a potent tool to design potential Scenarios in terms of environmental impacts, from which to learn how to improve productive processes and further reduce environmental impacts. This process can be done by observing in detail impact categories and the contribution of each process activity to the category impact to find solutions by combining individual technologies.^67^

The results discussed above indicate that the recovery of sewage sludge producing renewable fertilizers by AD allowed environmental benefits when the renewable fertilizers produced were used correctly and by efficient timing in substituting for synthetic mineral fertilizers, suggesting that the application of the Circular Economy in agriculture in terms of fertilization resulted in a win–win approach, which makes it more sustainable. However, as for all productive processes, impacts remain, and they cannot be nullified completely but only further reduced.

The detailed observation of every single impact, divided for impact categories and activities affecting each impact (Figure 3), allowed us to understand what are the more important factors in determining impacts. Emissions to air during field distribution of fertilizers (i.e., NH_3_ and N_2_O emission) seemed to affect greatly the ecosystem and human toxicity categories as they interacted with many impact subcategories (Figure 3a,b). Therefore, reducing air emissions allows the further reduction of an ecosystem and human impacts because of renewable fertilizer production and use. Digestate and ammonium sulfate produced by the plant studied in this work were used correctly following the best practice, i.e., digestate and ammonia injection, while the digestate was characterized by high biological stability, avoiding N mineralization and nitrate leaching. The strong impact reduction obtained by substituting synthetic mineral fertilizers with renewable fertilizers (Table 2 and Figure 2) confirmed this virtuous approach. Nevertheless, already stated, LCA can help in optimizing processes, further reducing impact.

**Figure 4:**
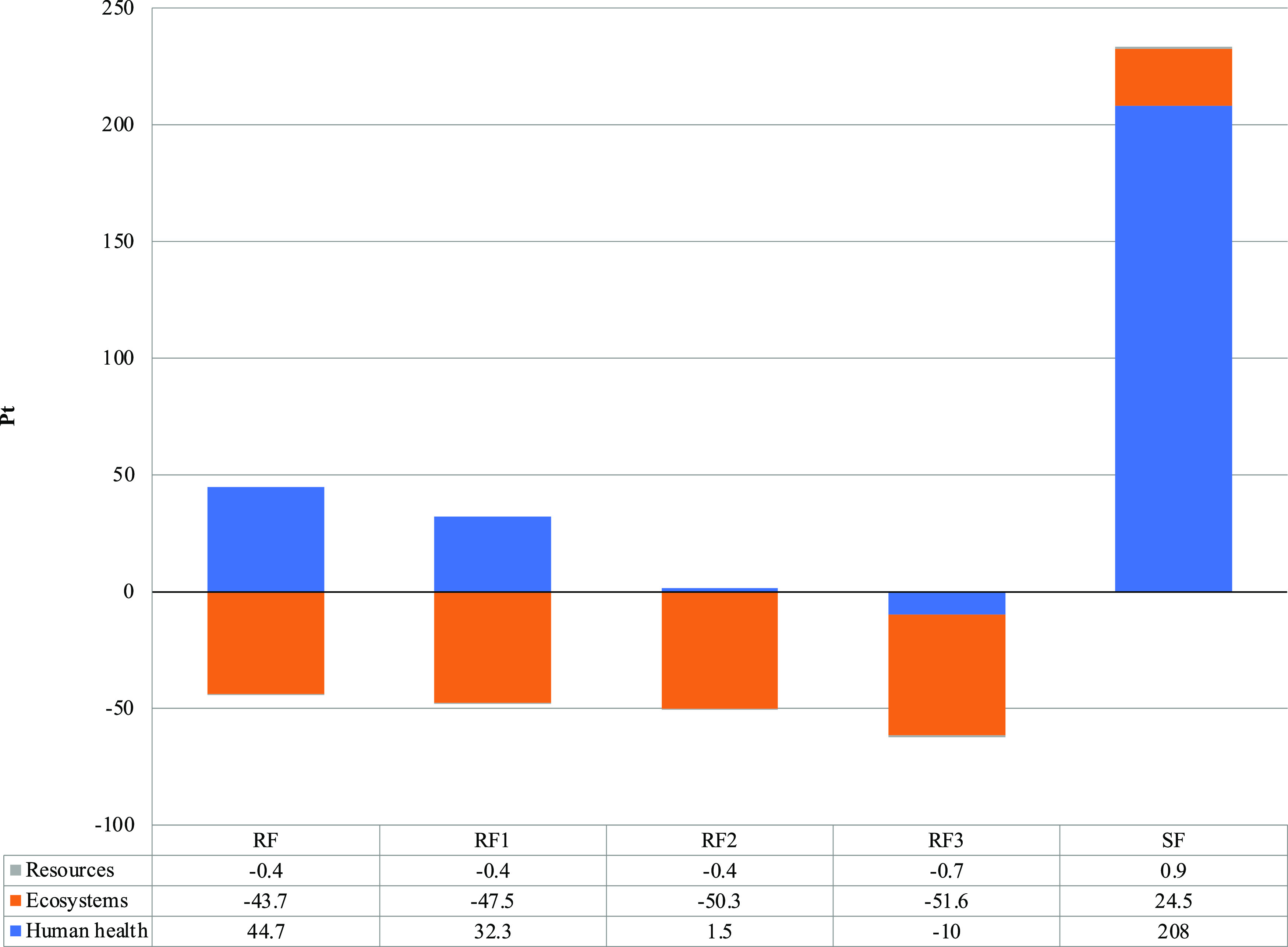
Comparative environmental results (Ecopoint—Pt) for the Scenario RF (recovered fertilizers), Scenario RF_1_ (RF + nitro inhibitor), Scenario RF_2_ (RF + nitro inhibitor + anchor), Scenario RF_3_ (RF + nitro inhibitor + anchor + biomethane for transportation), and Scenario SF (synthetic fertilizers). Impact assessment was calculated according to the ReCiPe 2016 end point (H) V 1.03 method.

Nitrogen dioxide emissions have been reported to be greatly reduced using nitrification inhibitors (NI).^68,69^ From the literature, it was calculated, on average, that the use of NI allowed a reduction of 44% in total N_2_O emissions,^70^ further reducing total Scenario RF impacts (Scenario RF_1_), with reference to ecosystem and human health impacts (Figure 4), if these data are implemented in the LCA. The modeling of this Scenario considered just the addition of NI to the soil reducing N_2_O emission (data from literature).^66^ The production (dicyandiamide) and distribution of the nitrohinibitor were considered negligible because of the very limited amount of product used (ca. 7 kg ha^–1^). In doing so, all of the data describing the Scenario remained the same as the original one (Scenario RF).

On the other hand, total ammonia emitted during digestate distribution can be reduced by optimizing the injection system. Preliminary data coming from work performed at full scale at the AD plant studied in this work indicated that by modifying the distribution equipment, i.e., Vervaet Terragator equipped with flexible anchors and a roller postposed to the anchors, allowed a reduction of ammonia emission of 44% (data not shown). The future integration of this practice will allow a further reduction of impacts, as shown in Figure 4 (Scenario RF_2_). Because the anchor system was applied to the digestate distribution system already in use, the only change in the Scenario modeling was referred to the emission of ammonia measured.

Another important activity that plays an important role in determining impact is transport. Transport affected a lot the terrestrial ecotoxicity (Figure 3b) and, although much less severely, many other subcategories within ecosystem and resources categories (Figure 3a,c) because of the fossil fuel used. Today, in the EU, anaerobic digestion represents a well-consolidated bioprocess treating organic wastes and dedicated energy crops, producing biogas/biomethane.^71^ In the Lombardy region alone, about 580 AD plants are operating producing biogas and now are starting to produce biomethane.^72,73^ Recently, a particular interest has been devoted to liquid biomethane (Bio-LNG) as a substitute for fossil fuels in truck transportation,^74^ and the first plants have started operating in Lombardy region, very close to the AD plant studied in this work. A new Scenario was modeled (RF3) assuming the biogas production from organic wastes (OFMSW and sludge), the purification and compression of biomethane, and the transport by 30 ton trucks and average consumption of fuel equal to 0.34 kg LNG per kilometer traveled.^75^ Emissions from trucks were recalculated accordingly.

Assuming an ability to substitute all fossil fuels with Bio-LNG produced from the organic fraction of municipal solid waste (Table 1) for transportation, a further strong impact reduction was obtained, nullifying completely the environmental impacts due to production and use of recovered fertilizers (Scenario RF_3_) (Figure 4).

## Conclusions

Nutrient recovery from organic waste represents a great opportunity to design a new approach in crop fertilization in the framework of the Circular Economy. Nevertheless, recycling nutrients is not enough, as recovered fertilizers should be able to substitute synthetic mineral fertilizers that contain high nutrient concentrations with high nutrient efficiency. A previous paper of ours^10^ reported that RF could be effectively obtained thanks to AD and that these RFs were good candidates for replacing SF. In this paper, the LCA approach indicates that producing and using those RFs instead of producing and using SF led to a strong environmental impact reduction. This result was due above all to the AD process that makes all this possible because of renewable energy production and biological processes modifying the fertilizer properties of digestate. Nevertheless, a correct approach in using RF is mandatory to avoid losing all of the advantages of producing RF because of impacts derived from incorrect RF use. In this way, a well-performed AD process assuring high biological stability of digestate, limiting RF-N_2_O emission and RF-NO_3_^–^ leaching, and RF injection limiting NH_3_ emissions, as well as using RF at the right time and according to crop requirements should be assured.

## Supporting Information Available

Full-field trial experiments and fertilization plan adopted; statistical approaches for data elaboration; fertilizer characteristics; emissions due to the use of fertilizers; heavy metal contents in soil and plant before and after fertilization; and crop yields (PDF)
