# Supplementary material for: Environmental Performance in the Production and Use of Recovered Fertilizers from Organic Wastes Treated by Anaerobic Digestion vs Synthetic Mineral Fertilizers
Source: ACS Sustain Chem Eng. 2022 Jan 7;10(2):986–97. doi: 10.1021/acssuschemeng.1c07028 (PMC8785226; doi:10.1021/acssuschemeng.1c07028)
Supplement: Supplementary file 1 — sc1c07028_si_001.pdf [file sc1c07028_si_001.pdf]

Supporting information for:

Environmental performance in the production and use of recovered fertilizers from organic wastes treated by anaerobic digestion vs. synthetic mineral fertilizers

Axel Herrera[1], Giuliana D'Imporzano[1*], Massimo Zilio[1], Ambrogio Pigoli[1], Bruno Rizzi[1], Erik Meers[2], Oscar Schouman[3], Micol Schepis[4], Federica Barone[4], Andrea Giordano[4], Fabrizio Adani[1**]

[1]Gruppo Ricicla - DiSAA, Università degli Studi di Milano, Via Celoria 2, Milano, Italy

[2]Department of Green Chemistry and Technology, Faculty of Bioscience Engineering, University of Ghent, Coupure Links 653, 9000 Ghent, Belgium

[3]Alterra, Part of Wageningen UR, PO Box 47, 6700 AA Wageningen, the Netherlands

[4]Acqua & Sole s.r.l., Via Giulio Natta, 27010 Vellezzo Bellini, PV, Italy

[*]Corresponding author e-mail address: giuliana.dimporzano@gmail.com

[**]Corresponding author e-mail address: fabrizio.adani@unimi.it

Number of pages: 9

S1. Additional material and methods

S2. Additional results.

Number of tables: 6

S1. Additional material and methods

Full Field trials

Data related to agronomic use of fertilizers in the two systems, RF and SF came from fertilization trials performed in the seasons 2018-2020. Fertilizers were tested on plots of 350 m^2 cropped with maize in 3 replicates, using a randomized experimental scheme. Thesis included the use of digestate from organic wastes combined with digestate-derived mineral fertilizer (ammonium sulphate) (RF) (Table S1 and S2) vs. synthetic fertilizers (SF), consisting in urea (N 46 %) and ammonia sulphate (21%) such as reported in the Table S3. In addition, to complete fertilization, 44.82 kg K Ha^{-1} as K_2O (KCL, 60 % K) was added to digestate plots, and 39.3 kgP Ha^{-1} as 0/46/0 complex and 69.4 kg K Ha^{-1} (KCl, 60 % K) were added to SF plots.

An unfertilized treatment was included as control. Digestate was distributed at pre-sowing by injection into the soil at a depth of 15 cm.

Statistical approach

The statistical analyses were carried out using IBM SPSS® 23 software. Determination of significant differences among the parameters analyzed at a level of significance of $P < 0.05$ was carried out by two-way analysis of variance (ANOVA) and Tukey's test.

S2. Additional results.

Crops yield

The crop yield was statistically no different between the RF and SF theses in the 3 years (Table S6)

Table S1. Main characteristics of infeed) and full characterization of digestate in comparison with legal limits for its use as fertilizer in agriculture, and with data from literature for digestate and composts (mean of three-years monitoring, from Pigoli et al., 2021)

Parameter	Unit	Digestate[a]	Lombardy Law N. 6665/2019 – Legal limits[b]
pH		8.5 ± 0.3	5.5 < pH < 11
Dry Matter 105°C	g kg^{-1} ww[c]	103 ± 3.7	
Dry Matter 600°C	g kg^{-1} ww	40.4 ± 2.5	
Total Organic Carbon	g kg^{-1} DM[c]	314 ± 30	> 200
TKN	g kg^{-1} DM	77 ± 3.7	> 15
$N-NH_4$	g kg^{-1} DM	35.9 ± 2.4	
$N-NH_4$/TKN	%	46.6	
OD_{20}[d]	mg O_2 g^{-1} DM	22.6 ± 6.1	
BMP[e]	L_{biogas} kg^{-1} DM	57 ± 23	
P	g kg^{-1} DM	28 ± 4.1	> 4
K	g kg^{-1} DM	6.5 ± 1.3	
Ca	g kg^{-1} DM	43 ± 7	
Mg	g kg^{-1} DM	5.2 ± 0.6	
Fe	g kg^{-1} DM	26.2 ± 6.4	
Mo	mg kg^{-1} DM	10 ± 1	
Cu	mg kg^{-1} DM	408 ± 60	≤ 1,000
Zn	mg kg^{-1} DM	1,020 ± 120	≤ 2,500
Mn	mg kg^{-1} DM	444 ± 35	
Al	g kg^{-1} DM	25.8 ± 4.5	
Co	mg kg^{-1} DM	6.6 ± 2.3	
Se	mg kg^{-1} DM	3.7 ± 2.1	≤ 10
Na	g kg^{-1} DM	1.9 ± 0.4	
Cr	mg kg^{-1} DM	95 ± 22	< 200
Pb	mg kg^{-1} DM	64 ± 11	≤ 750
Ni	mg kg^{-1} DM	61 ± 13	≤ 300
As	mg kg^{-1} DM	9.0 ± 2.2	< 20
Cd	mg kg^{-1} DM	1 ± 0.5[f]	≤ 20
Hg	mg kg^{-1} DM	0.1 ± 0.3[f]	≤ 10
PAH	mg kg^{-1} DM	0.5 ± 0.5[f]	∑< 6
PCB	mg kg^{-1} DM	< 0.1	∑< 0.8
PCDD/F+PCB-DL	ng TEQ kg^{-1} DM	10.6 ± 2.9[f]	∑≤ 25

DEHP	mg kg^{-1} DM	5.7 ± 5.3[f]	< 100
Hydrocarbon C10-C40	mg kg^{-1} ww<br>mg kg^{-1} DM	284 ± 251[f]<br>(2,757)	≤ 1,000
AOX	mg kg^{-1} DM	< 0.6	∑ < 500
Ciproflaxacin	mg kg^{-1} DM	< 0.01[g]	
Sulfamethoxazole	mg kg^{-1} DM	< 0.01	
Fenofibrat	mg kg^{-1} DM	< 0.01	
Gemfibrozil	mg kg^{-1} DM	< 0.01	
Carbamazepine	mg kg^{-1} DM	< 0.01	
Metoprolol	mg kg^{-1} DM	< 0.01	
Diclofenac	mg kg^{-1} DM	< 0.01	
Ethinylestradiol	mg kg^{-1} DM	< 0.01	
Estradiol	mg kg^{-1} DM	< 0.01	
Salmonella	MPN g^{-1} DM	Absent	< 100
Faecal coliform	MPN g^{-1} DM	< 1,000	< 10,000

[a]Mean ± SD: *n*=42, except for Ca, Mn, Mg, Fe, Mo, Al, Co, Na: *n* = 9, and BMP: *n* = 10.

[b]Legal limit referred to the digestate described in this work.

[c]ww and DM: wet weight and dry matter, respectively.

[d]OD_{20}: Oxygen Demand after 20h

[e]BMP: potential biogas production.

[f]Mean and SD calculated considering data below detection limits = 0.

[g]Analysis performed in 2020; *n*=4.

Table S2. Main characteristics of ammonium sulphate - $(NH_4)_2SO_4$ - derived from digestate, used in field trials (mean three years ± SD, n=17).

Parameter	Unit	Value
pH	pH	6.8 ± 1.3
EC	mS cm^{-1}	119 ± 27<br>(1:2.5 v/v 25 °C)
Dry Matter 105°C	% of ww	35.5 ± 0.4
Total Organic Carbon	g kg^{-1} ww	< 0.1
Total N	g kg^{-1} ww	74 ± 2
$N\text{-}NH_4$	g kg^{-1} ww	71.7 ± 1.9

Table S3. Main information regarding fertilization plan adopted: fertilization date, fertilizers used, and dose applied (RF = Recovered Fertilizers and SF = Synthetic Fertiliser).

Period	Plots	Fertilization	Fertilizer	Ntot applied (kg N ha^{-1})	Efficient N applied[a] (kg N ha^{-1})	Type of spreading
2018 - 2020	RF	Pre-sowing	Digestate	370	185	Injection 15 cm
		Top-dressing	Ammonia sulphate	100	100	Fertigation
	SF	Pre-sowing	Urea	185	185	Spread in surface
		Top-dressing	Ammonia sulphate	100[b]	100[b]	Fertigation

[a]Data calculated taking into consideration N efficiency for digestate of 0.5 and for urea of 1, according to Regional Plan for Water Protection from Nitrate from Agriculture (Regione Lombardia, 2020).

[b]On 2020: 90 kgN ha^{-1}

Table S4. Comparison between emissions (Ammonia, GHG and Nitrate leaching) and grain production measured from experimental soils fertilized with digestate and urea during the agronomic season (maize) (RF = Recovered Fertilizers and SF = Synthetic Fertiliser). The column "unfertilized" refers to the control plots set during the experimental design.

Parameter	Unit	RF	SF	Unfertilized
NH_3[a]	kgN ha^{-1}	25.6 ± 9.4(a)[b]	24.8 ± 8.3(a)	Undetectable[c]
N_2O[d]	kgN ha^{-1}	7.59 ± 3.2(ab)	10.3 ± 6.8(b)	1.71 ± 1.1(a)
CO_2[d]	kgC ha^{-1}	6216 ± 1160(a)	6144 ± 1491(a)	5698 ± 935(a)
CH_4[d]	kgC ha^{-1}	0036 ± 0.03(a)	0.053 ± 0.04(a)	0.066 ± 0.06(a)
NO_3^-[e]	mgN kg^{-1}	6.45 ± 7.6(a)	7.24 ± 8.6(a)	6.23 ± 7.1(a)
Grain Yield	Mg ha^{-1} DM[f]	18.1 ± 2.9(b)	17.4 ± 1.2(b)	10.4 ± 3.5(a)

[a]Cumulative emissions measurements carried out up to 90 hours after spreading (n = 9). The measures were repeated for three consecutive years (2018-2019-2020). Total N dosed: 370 kgN ha^{-1} (Digestate), 185 kgN ha^{-1} (Urea) (from Zilio et al., 2021)

[b]Letters in brackets are referred to One-way ANOVA analysis carried out for each of the emission source reported in the table (Tukey post-test, $p < 0.05$; $n = 3$).

[c]Ammonia emission in unfertilized plots did not differ from background.

[d]Cumulative emissions measurements carried out from 28/05/2020 (spreading) to 17/03/2021 (293 days, n = 36). Total N dosed: 370 kgN ha^{-1} (Digestate), 185 kgN ha^{-1} (Urea)

[e]Average concentration of NO_3^- in the soil at 1-meter depth. The measurements were carried out in 3 moments of the season (before spreading in pre-sowing, 20 days after spreading and after harvesting). n for each measure = 3. Total N dosed: 370 kgN ha^{-1} (Digestate), 185 kgN ha^{-1} (Urea)

[f]DM = dry matter

Table S5. Heavy metal content in soil before the pre-sown fertilization on March 2018 and after the end of the third crop season on January 2021, and Zn content in grain on 2020.

Parameter		Unit	March 2018	January 2021		
				Unfertilized	Synthetic fertilizer	Recovered fertilizer
As		mg kg^{-1} dw[a]	19.9 ± 1.1[b] (a)[c]	22.9 ± 2.8 (a)	19.6 ± 0.5 (a)	21.1 ± 2.3 (a)
Cd		mg kg^{-1} dw	<0.5	<0.5	<0.5	<0.5
Hg		mg kg^{-1} dw	<0.5	<0.5	<0.5	<0.5
Cr		mg kg^{-1} dw	39.2 ± 2.3 (a)	42.6 ± 2 (a)	40 ± 4.1 (a)	40.2 ± 1.6 (a)
Ni		mg kg^{-1} dw	23.3 ± 2.3 (a)	25.7 ± 1.7 (a)	25.9 ± 3.7 (a)	26 ± 1.6 (a)
Pb	Soil	mg kg^{-1} dw	32.8 ± 0.1 (a)	34.2 ± 4.2 (a)	33.4 ± 2.2 (a)	33.6 ± 4.5 (a)
Cu		mg kg^{-1} dw	19.1 ± 1.3 (a)	22.2 ± 3.3 (a)	21.4 ± 3.5 (a)	24.4 ± 3.1 (a)
Zn		mg kg^{-1} dw	69.8 ± 0.5 (a)	71.4 ± 3 (a)	71.4 ± 1.3 (a)	70.8 ± 1.8 (a)
Zn	(Grain)	mg kg^{-1} dw_{grain}		26.2 ± 3.67	25.0 ± 1.98 (a)	32.1 ± 1.9 (b)

[a]dw: dry weight

[b]mean ± SD; n=3

[c]Letters are referred to One-way ANOVA comparing values in each row (p<0.05; n=3; Tukey post-test).

Table S6. Average maize productions yield in grain for the three years of experiments (mean ± SD; n=9). Table modified from Zilio et al., 2021.

**Fertilizer**	**Grain yield dw[a] ($Mg\ Ha^{-1}$)**
Unfertilized	10.4 ± 3.5 (a)[b]
Synthetic fertilizer	17.4 ± 1.2 (b)
Recovered fertilizer	18.1 ± 2.9 (b)

[a]dw: dry weight

[b]Letters are referred to One-way ANOVA analysis (Tukey post-test, $p < 0.01$; n=9).
